# Practical Notes and Formulæ

**Published:** 1878-05-20

**Authors:** 


### PRACTICAL NOTES AND FORMULÆ.

### DIARRHŒA.

Dr. W. McWilliams, of Steam Corners, Ohio, says, in the *Ohio Medical Record*, August, 1877:

In the hot season, the common autumnal or summer diarrhœa is one of the most common complaints for which the physician has to prescribe. As most physicians are aware that a vast amount of it is caused and kept in action by an acid state of the stomach and indigestion, and that laxatives and antacids will generally control it, I present the simple and inexpensive form which I have employed for the last five years with entire success. It will sometimes, though rarely, be necessary to employ a little hydrargyrum cum creta or quinine in connection with it:

R.—Pulv. rhei.

Magnesiæ,

Sodii bicarb.................... aa grs. xl.

Sacchari alb.......................... oz. ij.

Ol. anisi.................................. gtt. xl.

Aquæ..................................... f. dr. viij.

Tinct. opii camph.................. f. oz. ss.

Drop the oil of anise on the sugar in a mortar, add the powders, and mix gradually; add the water, pour all into a bottle, and add the paregoric. Shake well before using.

Dose for infant, one-half a teaspoonful; one to two years old, one teaspoonful; two to ten years old, two teaspoonfuls; adults, one to two tablespoonfuls, from three to six hours apart. If it should be necessary to use an astringent, as dry chalk,

R.—Cretæ prep.......................... oz. j.

Pulv. kino.............................. dr. j.

M. May be prescribed in doses sufficient to produce the desired effect.

### HYPOSULPHITE OF SODA.

A. B. Loving, M. D., writes: I see from a number of your journal that some M. D. says that the hyposulphite of soda is almost gone out of use by the profession. "Be it far from me." I know of nothing that will relieve what is commonly known as chicken pox as quickly as the following prescription:

R.—Hyposulphite soda.................... dr. ii
Glycerine, or simple syrup, aqua, aa...oz. iss.

M. Teaspoonful every 2 or 3 hours. I have given the above prescription with advantage where one crop of boils would come out after another.

### QUARTAN INTERMITTENTS.

Dr. Baskersville (Horn Lake, Miss.), writes to *Med. & Surg. Rep.* that he has found the following prescription very efficient in all miasmatic disorders:

R.—Strychniæ sulph.................. grs. jss.

Quinæ sulph........................... dr. jss.

Ferri sulph. excicat.................. dr. j.

Acid sulph. aromat.................. dr. j.

M. And add—

Acidi arseniosi........................ gr. ij.

Podophyllin............................. grs. x.

Gelsmin.................................. grs. xx.

M. Make pills No. xc. Take one pill three times a day after meals.

These he omits on the chill day, and gives six grains of quinine every two hours until three or four doses are taken.

### NIGHT SWEATS.

Prof. Bartholow, in Good Samaratan Hospital (reported in 'The Clinic), recommends for night sweats the following:

R.—Strychniæ sulph.................. gr. j.
Atropiæ sulph....................... grs. 1-6
Morph. sulph......................... grs. vj.
Acid sulphuric dil.................. dr. j.
Aquæ distil........................... dr. vij.

M. Ten drops night and morning in water.

### SALINE APERIENT.

R.—Sodii sulphatis........................ oz. j.
Potassii sulphatis..................... dr. j.
Potassii bicarbonat...................... dr. j.
Lithii carbonat............................ grs. xv.

M. Teaspoonful in a glass of water before breakfast. Used by Prof. Duhring as a cooling aperient in eczema, and especially useful as a laxative in constipated habits attended with acid dyspepsia.

### APPROXIMATE MEASUREMENTS.

The following table gives an approximate estimate of the measurements in ordinary use; yet it shonld be remembered that they are not safe when applied to the more active or potent drugs:

| | | |
|---|---|---|
| Teaspoonful | 1 | fluid drachm. |
| Tablespoonful | 4 | do |
| Wineglassful | 2 | f. oz. |
| Tumblerful | 8 | do. |
| Thimbleful | ¾ | drachm. |
| Handful | 10 | do. |

### WHOOPING COUGH.

The following, improvised in the presence of patient, recently relieved a very obstinate case of whooping cough in a teething child. The case yielding all of its violence in three or four days:

R.—Whisky,

Water.............................. aa oz. ij.

Brom. potass........................ grs. xvj.

Tinc. assafœtida........................ dr. j.

Tinc, belladonna......................: gtt. v.

M. Dose, one teaspoonful to a child 8 months old, every 2 to 4 hours. W.

### WEATHERLY'S FAMOUS CATARRH CURE.

The above may be prepared as follows:

R.—Loaf sugar............................ 2 parts.
Borax
Common sslt each..................... 1 part.
Oil of peppermint a few drops.
Triturate.—*New Rem.*

### PENTHORUM SEDOIDES

Is a California plant used in diseases of the mucous membranes. In nasal catarrh, in lucorrhea, gleet, etc. Dose, 15 drops three times per day.

### ERGOTIN HYPODERMICALLY.

R.—Ergotin ....................................gr. ij.

Spiritus vini rectificati

Glycerine puræ..... ...... ......aa dr. ss.

M. Dose v. minims, equal to gr. 1-6.

### CORROSIVE SUBLIMATE HYPODERMICALLY.

R.—Corrosive sublimate ..................gr. ili.

Morphæ sulphatis ...................gr. iss.

Aqua distillatæ ........................oz. iij.

M. Fifteen minims—1-32 of a grain, will suffice for a single injection.

### ATROPIA HYPODERMICALLY.

The dose of the sulphate of atropia, hypodermically, is suggested in the following methods: gr. ¼ to f. dr. i., of which 5 minims, equivalent to 1-48 of a gr.; gr. ½ to f. oz. i., of which 2½ minims, or 1-48 of a gr., gr. 1 to f. dr. i., of which 1 minim, equal to 1-60 of a gr. This preparation is rarely used, except in opium poisoning.

### NOCTURNAL PAINS.

The nocturnal pains of tertiary syphilis may be relieved by the following:

R.—Sulph. morph.
Sulph strychnia....................aa gr. ss.
Bromide of calcium ........ ..........dr. j.
Syrup.
Peppermint water .................aa oz. iij.

M. One tablespoonful at bedtime, repeated if necessary.—(Dr. Maury, in *Med. and Surg. Rep.*

### PURGATIVE POWDER.

R.—Podophylin ..........................grs. v.
Leptandrin................................grs. vj.
Hydrarg. cum creta..................grs. xl.

M. Triturate thoroughly and give two to three grains at a dose. An efficient laxative or purgative, according to the dose used, well suited to hepatic disorders, and admirally adapted to the initia stages of bilious fever, dysentery, etc.

### SUBCUTANEOUS MEDICATION.

According to Dunglison, the best menstrum, as a general rule, is distilled water, and great care should be taken that the solution is perfect and free from foreign substances either acid or alkaline; should be fresh when used and filtered.

### NEURALGIC REMEDY.

Surgeon General Frances, of the British army, states that many cases of neuralgia which resist quinine and arsenic and seem incurable, only require large doses to effect a cure. In India he has been in the habit of prescribing, with almost unfailing success, ten, twenty and thirty grains of quinine, and when this has failed he has given as much as twenty to thirty minims of Fowler's solution of arsenic successfully. Sometimes the two remedies were combined as follows:

| | |
|---|---|
| R.—Sulph. quin | 10 grains. |
| Fowler's solution | 15 drops. |

Three times per day until the habit is broke, and then continued a few days in reduced doses.

### ARSENIC IN MANIA.

An old and somewhat obscure physician of our acquaintance, has acquired some celebrity for curing crazy people, many of whom have been sent to him from a distance. Upon inquiry he informed us that his remedy was arsenic—Fowler's solution in small doses long continued.

### CREOSOTE IN ULCERS.

Creosote may be applied with advantage to ulcers of various kinds; but in the scrofulous, aphthous, phagedenic and venereal kinds it has been found most useful.

The following wash is spoken of with great favor as having been used in a case of aphthous ulceration of the mouth:

| | |
|---|---|
| R.—Creosote | ½ drachm. |
| Gum acacia | 2½ ounces. |
| Mist. camphoræ | 11½ ounces. |

M.—*Brief.*

### ANTI INSECTS.

A correspondent of the Germantown *Telegraph* writes: Melon and cucumber bugs like radish leaves better than any other kind. I sow a few radish seeds in each hill and never lose a plant. Earth worms, cut worms, white grubs, and in fact all soft-bodied worms, are easily driven out by salt sowed broadcast. You can do no harm with ten bushels to the acre, but a half bushel is ample. Dry slaked lime is also effectual.

### SCALP WOUNDS.

In two cases recently, we tried, in lieu of sutures, tying strans of the hair so as to bring the lips of the wound on the scalp in opposition. The method was satisfactory. To prevent slipping it may be necessary to tie a series of knots, or to bind the knot with at hread. G.

### VALERIANATED SYRUP OF RHUBARB.

| | |
|---|---|
| R.—Syr. rhei | 4 ounces. |
| Tinct. valerian | 2 " |
| Oil of sassafrass | 20 drops. |
| Piperin | 10 grains |
| Sub. carb. of soda | 20 " |

M. This remedy has been highly extalled in typhoid fever as a nerve stimulant and to improve the secretions and make uniform the peristaltic motion in flatulent and tympanetic conditions. Dose, one tablespoonful three times per day.

A New Adhesive Plaster, especially adapted to the requirements of modern surgery, has been studied out by Dr. H. A. Martin, of Boston. It will be prepared by Messrs. Metcalfe & Co., who will present a specimen of the plaster to any physician who may apply, either personally or by letter. The ingredients of the plaster are the best Para rubber, Burgundy pitch, and balsam of tolu. The cloth undergoes an antiseptic treatment by liq. zinci chloridi before the compound is spread upon it.—*Proceed. County Kings.*

### STRYCHNIA.

"The last dose from a bottle containing a mixture of strychnia and bromide of potassium," says the Detroit *Medical Journal*, "poisoned the patient. The bromide had precipitated the strychnia." —*BostonMed. and Sur. Jour.*

